# REFGEN and TREENAMER: Automated Sequence Data Handling for Phylogenetic Analysis in the Genomic Era

**DOI:** 10.4137/ebo.s2331

**Published:** 2009-05-06

**Authors:** Guy Leonard, Jamie R. Stevens, Thomas A. Richards

**Affiliations:** 1 Centre for Eukaryotic Evolutionary Microbiology, School of Biosciences, University of Exeter, Geoffrey Pope Building, Exeter, EX4 4QD, U.K; 2 School of Biosciences, University of Exeter, Hatherly Laboratories, Exeter, EX4 4PS, U.K

**Keywords:** phylogeny, branch labels, sequence alignment, text management

## Abstract

The phylogenetic analysis of nucleotide sequences and increasingly that of amino acid sequences is used to address a number of biological questions. Access to extensive datasets, including numerous genome projects, means that standard phylogenetic analyses can include many hundreds of sequences. Unfortunately, most phylogenetic analysis programs do not tolerate the sequence naming conventions of genome databases. Managing large numbers of sequences and standardizing sequence labels for use in phylogenetic analysis programs can be a time consuming and laborious task. Here we report the availability of an online resource for the management of gene sequences recovered from public access genome databases such as GenBank. These web utilities include the facility for renaming every sequence in a FASTA alignment file, with each sequence label derived from a user-defined combination of the species name and/or database accession number. This facility enables the user to keep track of the branching order of the sequences/taxa during multiple tree calculations and re-optimisations. Post phylogenetic analysis, these webpages can then be used to rename every label in the subsequent tree files (with a user-defined combination of species name and/or database accession number). Together these programs drastically reduce the time required for managing sequence alignments and labelling phylogenetic figures. Additional features of our platform include the automatic removal of identical accession numbers (recorded in the report file) and generation of species and accession number lists for use in supplementary materials or figure legends.

## Introduction

Sequence based phylogenetic methods can be powerful techniques that enable the reconstruction of gene, cell and species evolution. With increasing access to gene and genomic data the collection of sequences for phylogenetic analyses can be a time-consuming process and can involve multiple sequence similarity searches (e.g. BLAST searches)^1^ of numerous databases. It is advisable to follow this process in order to control for both taxon and paralogue sampling issues and to break long branches (for examples and discussion see).^2^–^5^ Therefore, phylogenetic analyses can often begin with several hundred sequences. We find that most sequences are returned from gene databases, for example GenBank (http://www.ncbi.nlm.nih.gov/), in a labelled format incompatible with phylogenetic analysis programs. This process therefore requires re-labelling of sequence names in an alignment file, often with the need to rename the subsequent phylogenetic tree figure. If a phylogenetic analysis includes many hundred sequences and requires multiple taxon optimisation steps, this process can be very time consuming. Here we report the availability of a beta version of an online suite of programs for the management of database sequence data and phylogenetic tree files in order to expedite the process of sequence sampling, management of sequence names in alignments, and labelling of phylogenies.

## Methods

**Figure 1:**
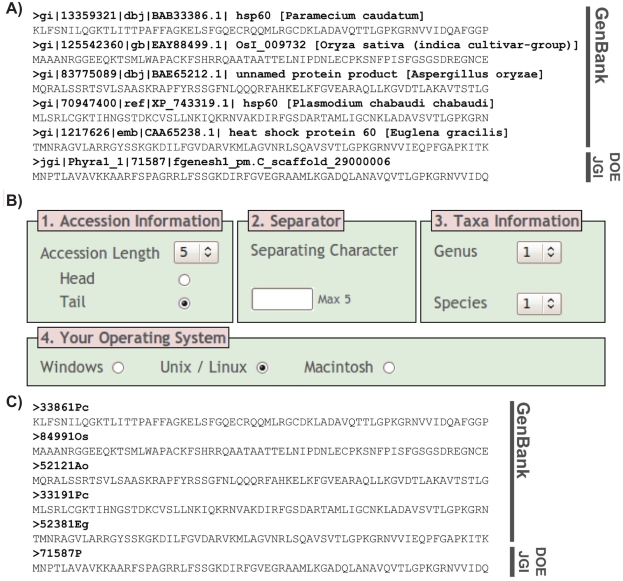
REFGEN conversion of FASTA files for use in phylogenetic programs. **A)** Snapshot of CPN60 alignment. Sequences are derived from GenBank and the DOE JGI *Phytophthora ramorum* databases (please note although the DOE JGI sequence does not confirm to the long identification line format it is accommodated by REFGEN). All CPN60 sequences are curtailed after the first 70 amino acid positions for the purpose of this figure. Note the long database identifier lines given to each sequence. **B)** Screenshot of REFGEN formatting options. **C)** Output from REFGEN, with sequence labels now compatible with all phylogenetic programs and ready for analysis.

Prior to any phylogenetic analyses, it is often necessary to conduct a series of BLAST searches to sample gene sequences from GenBank and other online databases (e.g. Department of Energy Joint Genome Initiative—http://genome.jgi-psf.org). It is then necessary to collect the returned FASTA formatted sequences in a single alignment file. As standard in the GenBank database and other data repositories, each database sequence has been given a long identification line (Fig. 1A) also known as the header or definition line. Within each sequence identification line the accession number and species name is recorded (species names are generally contained within square brackets). Our first program REFGEN (http://www.exeter.ac.uk/ceem/refgen.html) will take an uploaded FASTA sequence alignment file and convert each long sequence identification line, according to a set of renaming rules in to a REFGEN ID (Fig. 1C). The REFGEN ID is comprised of a combination of either the taxonomic name or the accession number (Fig. 1B). In some cases this may generate two or more identical REFGEN IDs, in these cases the last character of the identical REFGEN IDs will be removed and the REFGEN ID will be appended with A, B, C, etc. Where no species name exists in the long identification line, text from within the parentheses is used, and where this is not found the first two words in the locus are recorded. REFGEN will then output a FASTA sequence file with the new shortened names (REFGEN IDs) that are compatible with all phylogenetic analysis programs. The great majority of GenBank protein sequences have the standard header/definition line format; this is also the case, but to a lesser extent, for many GenBank nucleotide sequences. However, there are exceptions and we have therefore specifically designed the program so that any sequence data that does not follow this format (e.g. many JGI sequences) are accommodated by being appended untouched to the end of the results file for direct user evaluation.

Two additional files are provided by REFGEN, first a key ‘species list’ for use with downstream operations and a list of any duplicated sequences that have been removed. We find that during the process of BLAST search sequence sampling, especially when paying attention to paralogue sampling, often a database sequence can be sampled more than once. REFGEN will remove sequences with identical database long identification lines and record such removals within the ‘species list’. Second, a ‘log file’ contains all REFGEN alterations so sequences that are not preformatted with database long identification lines read by REFGEN can be traced and manual alterations performed accordingly. The sequence file is now ready for alignment, model evaluation (we suggest MODELGENERATOR)^6^ and phylogenetic analyses.

**Figure 2:**
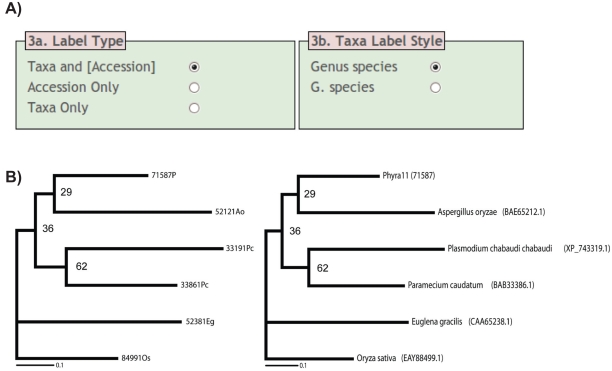
TREENAMER conversion of phylogenetic analysis with REFGEN IDs. **A)** Screenshot of TREENAMER tree formatting options. **B)** Example of tree output, the leftmost tree results from phylogenetic analysis. The rightmost tree is the same tree after editing with TREENAMER. Please note although the DOE JGI sequence does not conform to the long identification line format it is accommodated by TREENAMER, such sequences will require manual alteration in the final figure but can be easily traced using the REFGEN output files.

Post phylogenetic analyses, standard outputs (e.g. NEWICK or NEXUS tree files) from many phylogenetic analysis programs (e.g. PHYLIP,^7^ PHYML,^8^ MRBAYES,^9^ RAXML,^10^ and PAUP),^11^ can be uploaded with the relevant REFGEN ‘species list’ to TREENAMER. The TREENAMER (http://www.exeter.ac.uk/ceem/treenamer.html) program will then re-label all the branch names, replacing the REFGEN user defined sequence identifiers with a combination of full species names and/or accession numbers. The style of the branch labels can be user defined (Fig. 2A). In addition, a text (CSV) file is generated which includes a list of species with accession numbers used for the phylogenetic analyses. This file can be used for figure legends or supplementary materials. At this point some manual alterations are required if the original dataset includes sequences that do not conform to the standard header/definition line format. This process can be guided by the REFGEN ‘species list’.

The final output includes a fully labelled NEWICK tree file with the style of the branch labels specified by the user (Fig. 2A). These files can be drawn using TREEVIEW (http://taxonomy.zoology.gla.ac.uk/rod/treeview.html) or FigTree (http://tree.bio.ed.ac.uk/software/figtree/) (Fig. 2B). We have also designed the program so that if the process of phylogenetic analysis requires the removal of sequences from the alignment, it is possible to still use the original REFGEN ‘species list’ in combination with the resulting tree file to perform the TREENAMER step.

Both program scripts are implemented using the PERL language (www.perl.com) in conjunction with the CGI.pm module library (http://search.cpan.org/dist/CGI.pm/) that allows scripts to upload files to a web space via CGI. The extraction of accession numbers and species names is implemented with the use of regular expressions for pattern matching the FASTA formatted and NEXUS/NEWICK text files. This allows for easy integration of new and the modification of old formats of the FASTA definition lines specified by different databases. All the variables extracted using regular expressions are temporarily stored in arrays for better data manipulation. Information from the arrays is extracted and output online, generating files ready for download. These resources and the program scripts are accessed through a HTML front-end located on the CEEM website hosted by the University of Exeter. Generated data will be available for a minimum of one week from time of creation to preserve space on the server.

## Conclusion

Here we describe two web utilities REFGEN and TREENAMER which are available online at http://www.exeter.ac.uk/ceem/refgen.html and http://www.exeter.ac.uk/ceem/treenamer.html. These programs enable users to efficiently manage the naming of database sequences for alignment and subsequently branch labels of phylogenetic trees. Both steps can be user controlled so that a researcher can alter the standardised labelling for both the alignment and the subsequent tree files using a combination of taxon names and accession numbers. The programs also report a table of species names and database accession numbers for use in figure legends and supplementary materials and will remove identical database entries that may be included during database sampling. We note that although these tools have the capacity to radically reduce time requirement in preparing phylogenies, the tools are only highly effective when a large proportion of the sequences sampled follow the database long identification line format described above, for example, GenBank protein database. The online suite includes the ability to report any problems or suggest improvements. We hope this will provide a useful resource to the comparative genomics and phylogenetics communities.
